# Efficacy of Dexmedetomidine vs Morphine as an Adjunct in a Paravertebral Block with Bupivacaine in Postoperative Analgesia Following Modified Radical Mastectomy

**DOI:** 10.7759/cureus.8231

**Published:** 2020-05-22

**Authors:** Malik Jamil Ahmed, Abaid Ur Rehman, Rana Muhammad Arshad, Muhammad Wasim Ali Amjad, Zeeshan Khan, Aamir Furqan

**Affiliations:** 1 Anaesthesia, Bakhtawar Amin Medical and Dental College, Multan, PAK; 2 Anaesthesia, Lahore Medical and Dental College, Ghurki Trust Teaching Hospital, Lahore, PAK; 3 Anaesthesia, Services Institute of Medical Sciences, Lahore, PAK; 4 Anaesthesia, Sheikh Zayed Medical College and Hospital, Rahim Yar Khan, PAK; 5 Anaesthesia and Critical Care, Chaudhry Pervaiz Elahi Institute of Cardiology, Multan, PAK

**Keywords:** dexmedetomidine, morphine, paravertebral block (pvb), modified radical mastectomy (mrm)

## Abstract

Objective

To observe the efficacy of dexmedetomidine vs morphine as an adjunct in a paravertebral block (PVB) with bupivacaine in postoperative analgesia following modified radical mastectomy.

Study design

This was a randomized controlled trial performed from June 2018 to August 2019 in the Department of Anesthesia, Bakhtawar Amin Medical and Dental College, Ch. Pervaiz Ellahi Institute of Cardiology, Multan, Gurki Hospital, Services Institute of Medical Sciences, and Sheikh Zayed Hospital, Lahore.

Methodology

Seventy-eight patients were equally divided into group M, which received morphine (3 mg) and group D, which received dexmedetomidine (1 µg/kg), along with 20 cc 0.25% bupivacaine, for PVB. The primary outcome included morphine requirements in the post-anesthesia care unit (PACU). Secondary outcomes included the quality and duration of analgesia, intraoperative doses of fentanyl and propofol, postoperative doses of diclofenac required, postoperative nausea and vomiting (PONV), and the Ramsey sedation score. Data were entered into SPSS version 23 (IBM Corp., Armonk, NY) and analyzed by applying the independent t-test, Mann Whitney U-test, and the chi-square test or Fischer’s exact test, as appropriate. P≤0.05 was considered statistically significant.

Results

The mean time for the first analgesic administration was much shorter in group D as compared to group M (p<0.001). The average doses of ephedrine and morphine used were higher in group D (p-value 0.033 and 0.013, respectively). In the PACU, 33.3% of group D patients as compared to 12.8% of group M patients needed morphine (p=0.032). Postoperatively, diclofenac consumption was higher in group D (p<0.001). Postoperative pain was lower and sedation was higher in group M (p<0.05).

Conclusion

As an adjunct to bupivacaine in PVB for MRM, morphine is superior to dexmedetomidine.

## Introduction

Among women, breast cancer is the most common to require surgical intervention [1-2]. Usually, general anesthesia is used for conducting breast surgery but this is associated with a very high incidence of postoperative nausea and vomiting (PONV as well as severe pain [3]. This pain can be disadvantageous to patients’ recovery and health. There are many flaws associated with the postoperative pain experienced following mastectomy, and these flaws include prolongation of hospital stay, a poor recovery profile, and an increased chance of progression to chronic pain. A variety of techniques, including intercostal block, thoracic epidural anesthesia, local anesthetic infiltration paravertebral block, field block, brachial plexus block, and pectoral nerve blockage, have been adopted to reduce postoperative pain, avoid the complications of general anesthesia, and reduce the duration of hospital stay following mastectomy [4-5].

Spinal nerves, the sympathetic chain, gray and white rami communicantes, intercostal vessels, and fat are contained in a potential space known as the paravertebral space. When local anesthetic agents are injected into this space, a dense block is produced due to direct infiltration of the sympathetic chain as well as spinal nerves. But the majority of the local anesthetic agents provide very short analgesia, which warrants the use of various adjuvants that include clonidine, magnesium, epinephrine, tramadol, dexamethasone, opioids, and dexmedetomidine for enhancing the quality as well as the duration of the analgesia in a paravertebral block [6-7].

In patients undergoing breast cancer surgery under general anesthesia, very promising results have been observed with the use of 1 µg/kg dose of dexmedetomidine in addition to bupivacaine for a paravertebral block. A significant decrease in postoperative pain has been observed, along with reduced consumption of morphine and tramadol [8-9]. The efficacy of 2 mg morphine as an adjunct to 0.25% bupivacaine for a paravertebral block has also been studied with satisfactory outcomes [10-11]. According to a study, postoperatively, three times more additional opioids were required by patients who were given only general anesthesia, as compared to those who also received PVB [12]. We conducted this study with the purpose of comparing the efficacy of a paravertebral block with bupivacaine while using dexmedetomidine or morphine as an adjunct in reducing postoperative pain in female patients undergoing modified radical mastectomy with or without axillary clearance.

## Materials and methods

We conducted this randomized controlled trial after getting approval from the hospital ethics committee at the department of anesthesia at Bakhtawar Amin Medical and Dental College, Ch. Pervaiz Ellahi Institute of Cardiology, Multan, Gurki Hospital, Services Institute of Medical Sciences, and Sheikh Zayed Hospital, Lahore, from June 2018 to August 2019. The sample size was calculated from our reference study, which was performed by Megha T et al. With the nonprobability consecutive sampling technique, we selected 78 women of American Society of Anesthesiologists status I or II who were between 18 and 60 years of age and were planned to undergo modified radical mastectomy with or without axillary clearance due to breast carcinoma. All the patients who had an allergy to local anesthesia drugs, bleeding disorders, injection site infection, pregnancy, and psychiatric disorders or were breastfeeding women were excluded from our study.

All the patients were fully aware of the nature of the procedure, and the method to report the numerical rating score and PONV was explained to them. Written informed consent was taken before the commencement of the study. Preanesthetic evaluation of all the patients was performed, and ranitidine 150 mg and diazepam 0.15 mg/kg body weight were given to all the patients on the night prior to surgery. All the patients were randomly allocated into two groups with 39 patients each. Group M was given 20 cc of 0.25% bupivacaine and morphine 3 mg for PVB while group D received 20 cc of 0.25% bupivacaine along with dexmedetomidine 1 µg/kg for PVB. After securing the intravenous (IV) lines, intravenous fluid was given at 20 ml/kg/h till anesthesia induction and, later on, fluid was given after calculation according to body needs. Patients were monitored with a non-invasive blood pressure monitor, pulse oximeter, and electrocardiogram. Patients were in the lateral position with the side of the paravertebral block upwards. Identification and marking of the T3 vertebral spinous process were done. After strict aseptic measures, 5 cc of 1% lignocaine was infiltrated, 2.5 cm lateral to the cephalad edge of the spinous process of T3, into the skin, subcutaneous tissue, and periosteum of the T4 vertebral transverse process. At the site of local anesthesia, a 22G needle was inserted up to the transverse process and, after noting the depth, the needle was withdrawn and then again inserted caudal to pass by the transverse process for 1- 1.5 cm. The needle was placed at the site where the motor stimulation was best with a 0.6 mA current. After this, the respective drugs under study were injected in a 3-5 cc quantity with intermittent aspirations. A successful block was defined as the inability to feel a pinprick or cold sensations over the T1-T6 dermatomes after 20 minutes of drug infiltration. General anesthesia was induced and maintained with appropriate doses of vecuronium, propofol, fentanyl, and a 50% mixture of nitrous oxide and oxygen. An additional dose of fentanyl was given to control any clinically noted pain. Intravenous ephedrine was given to maintain the mean arterial pressure above 60 mmHg but no other analgesic was during the intraoperative period. In the PACU, morphine was given for analgesia while in the ward, diclofenac was the analgesic of choice. Ondansetron was given for controlling nausea and vomiting. The Ramsey sedation score (patient anxious=1; cooperative=2; response to command only=3; brisk response to light glabellar tap=4; lethargic response to light glabellar tap=5; no response=6), nausea and vomiting (0= no nausea and vomiting; 1= nausea present with no vomiting; 2= nausea and vomiting present), and pain with NRS score (0=no pain; 10=worst pain) were noted at 30 minutes, six hours, and 12 hours postoperatively.

The primary outcome included morphine requirements in the PACU. Secondary outcomes included quality of analgesia, analgesia duration, intraoperative doses of fentanyl and propofol, postoperative doses of diclofenac required, PONV, Ramsey sedation score, and any complication of PVB. All the data were collected by the researchers themselves. Data were entered in computer software SPSS version 23 (IBM Corp., Armonk, NY) and analyzed by applying the independent t-test, Mann Whitney U test, and chi-square test or Fischer’s exact test, as appropriate. P≤0.05 was considered statistically significant.

## Results

The data regarding mean age, body mass index (BMI), American Society of Anesthesiologists (ASA) status, and the side of surgery were comparable in group M and group D (p>0.05) (Table 1).

**Table 1:** Baseline data Data is mentioned as mean ± S.D unless mentioned otherwise. BMI: body mass index; ASA: American Society of Anesthesiologists

Variable	Group M (n=39)	Group D (n=39)	P-Value
Age, years	45.01 ± 6.04	43.64 ± 7.22	0.370
BMI, kg/m^2^	23/56 ± 3.19	23.26 ± 4.16	0.715
ASA-I/ASA-II	28 / 11	27 / 12	0.804
Side of surgery (right/left)	23 / 16	19 / 20	0.364

The duration of surgery was not statistically different between the two groups (p>0.05). The duration of anesthesia was 172.13 ± 24.20 min in group M and 154.02 ± 19.56 min in group D (p=0.001). The consumption of propofol and fentanyl was 712.26 ± 209.56 mg and 234.12 ± 51.98 µg in group M; and 565.56 ± 114.37 mg and 217.48 ± 42.12 µg in group D (p-value <0.001 and 0.108), respectively. The average doses of ephedrine and morphine used were 3.13 ± 2.22 mg and 1.04 ± 1.22 mg in group M; and 4.08 ± 1.57 mg and 1.81 ± 1.48 mg in group D (p-value 0.033 and 0.013), respectively. In the PACU, 33.3% of the patients in group D required morphine as compared to 12.8% of the patients in group M (p=0.032). The mean time for the first analgesic administration was much shorter in group D, i.e. 336.59 ± 154.88 minutes as compared to group M, i.e. 877.05 ± 161.79 minutes (p<0.001). Postoperatively, the dose of diclofenac used was 75.33 ± 13.59 mg and 179.46 ± 31.79 mg in groups M and D, respectively (p<0.001). The consumption of ondansetron in the postoperative period was higher in group M than in group D but the difference was not statistically significant (p=0.095) (Table 2).

**Table 2:** Intraoperative and postoperative data Data is mentioned as mean ± S.D unless mentioned otherwise. PACU: postanesthesia care unit

Variable	Group M (n=39)	Group D (n=39)	P Value
Surgery duration, min	142.21 ± 31.85	132.05 ± 33.15	0.172
Anesthesia duration, min	172.13 ± 24.20	154.02 ± 19.56	0.001
Propofol consumption, mg	712.26 ± 209.56	565.56 ± 114.37	<0.001
Fentanyl consumption, µg	234.12 ± 51.98	217.48 ± 42.12	0.108
Ephedrine dose, mg	3.13 ± 2.22	4.08 ± 1.57	0.033
Morphine consumption, mg	1.04 ± 1.22	1.81 ± 1.48	0.013
Number of patients receiving morphine in PACU, n (%)	5 (12.8)	13 (33.3)	0.032
Time for first analgesia, min	877.05 ± 161.79	336.59 ± 154.88	<0.001
Postoperative diclofenac consumption, mg	75.33 ± 13.59	179.46 ± 31.79	<0.001
Postoperative ondansetron consumption, mg	1.84 ± 0.98	1.49 ± 0.80	0.095

Postoperative pain was not different between the two groups 30 min postoperatively (p=0.123). At six and 12 hours, the pain was higher in group D as compared to group M (p-value 0.032 and <0.001, respectively). The Ramsey sedation score was higher in group M at 30 minutes, six hours, and 12 hours postoperatively as compared to group D (p<0.001). The incidence of PONV was not significantly different at 30 minutes and six hours postoperatively (p-value 0.152 and 0.210, respectively), but the incidence was higher in group M at 12 hours postoperatively (p=0.039) (Table 3).

**Table 3:** Postoperative pain, Ramsey sedation score, and nausea and vomiting Data is mentioned as median (IQR) unless mentioned otherwise PONV: postoperative nausea

Variable	Time	Group M (n=39)	Group D (n=39)	P-Value
Postoperative pain (0-10)	30 minutes	2 (1-2)	2 (1-3)	0.123
	6 hours	2 (1-2)	3 (1-3)	0.032
	12 hours	2 (2-3)	3 (2-3)	<0.001
Ramsey sedation score (1-6)	30 minutes	2 (2-3)	2 (1-2)	<0.001
	6 hours	2 (2-2)	1 (1-2)	<0.001
	12 hours	2 (1-2)	1 (1-2)	0.003
PONV (0/1/2)	30 minutes	37/0/0	39/0/0	0.152
	6 hours	36/2/1	39/0/0	0.210
	12 hours	33/4/3	39/0/0	0.039

## Discussion

In our study, we observed that the duration and quality of analgesia were significantly better in patients who received morphine along with bupivacaine for a paravertebral block. Also, the postoperative consumption of morphine and diclofenac was significantly lower in group M [13-15]. Our results were similar to the results observed by Megha T et al. [16]. In a couple of studies, it was observed that, in contrast to bupivacaine alone or no PVB, the use of dexmedetomidine as an adjuvant to bupivacaine for PVB in breast surgery prolonged analgesia while decreasing the morphine requirement and decreasing the incidence of PONV [16-17]. In another study, dexmedetomidine addition to bupivacaine for PVB increased the time for first rescue analgesia as compared to bupivacaine alone. Bhuvaneswari V et al., in their study, found the combination of bupivacaine, epinephrine, and fentanyl for PVB to be much better in terms of prolongation of postoperative analgesia [18]. However, the removal of fentanyl from the combination did not give good analgesic results.

Morsy AR et al. compared 0.25% bupivacaine, 0.25% bupivacaine with dexmedetomidine 100 µg, and 0.25% bupivacaine with 2 mg morphine in PVB executed with the landmark-guided method [10]. They observed better outcomes in patients who were given 0.25% bupivacaine with dexmedetomidine 100 µg as compared to other groups. These results were different from those of our study, i.e. morphine is better than dexmedetomidine, which might be due to the use of different doses of morphine (3 mg) and dexmedetomidine (1 µg/kg) in our study. Also, we used the nerve stimulation method for the execution of PVB. In a previous study, the nerve stimulation technique has been suggested to be much safer [18-19].

In our study, the dose of propofol used was higher in the morphine group as compared to the doses used in the dexmedetomidine group. The reason behind this has not been explained so far. However, in the past, the reduction in the intraoperative anesthetic drug, such as propofol requirement, has been reported when dexmedetomidine was used as an adjunct in the paravertebral block [20]. We observed a high incidence of postoperative nausea and vomiting in the patients who were given morphine as compared to the group that received dexmedetomidine. The reason behind this is that opioid derivatives have the tendency to induce emesis whereas dexmedetomidine has anti-emetic properties.

## Conclusions

As an adjunct to bupivacaine in a paravertebral block for modified radical mastectomy, morphine and dexmedetomidine were compared and the observations of our study revealed that among the studied variables, anesthesia duration, propofol consumption, ephedrine requirement, first analgesia demand, diclofenac consumption, postoperative pain, Ramsey's sedation score, and postop nausea and vomiting frequency, morphine is superior to dexmedetomidine in all except propofol consumption. So it can be concluded that morphine is a better choice as an adjunct with bupivacaine for a paravertebral block.
